# Over-the-counter medication use among Mexican immigrants in Southern Arizona: a cross-sectional study

**DOI:** 10.3389/fpubh.2025.1528486

**Published:** 2025-08-06

**Authors:** Estefania Ochoa Mora, Adriana Maldonado, Melissa Flores, Mariella Rodríguez, Daniel E. Martínez, Rebecca Crocker, David O. Garcia

**Affiliations:** ^1^Clinical and Translational Sciences Program, School of Health Professions, Mel and Enid Zuckerman College of Public Health, University of Arizona, Tucson, AZ, United States; ^2^Department of Health Promotion Sciences, Mel and Enid Zuckerman College of Public Health, University of Arizona, Tucson, AZ, United States; ^3^Department of Psychology, College of Science, University of Arizona, Tucson, AZ, United States; ^4^School of Sociology, College of Social and Behavioral Sciences, University of Arizona, Tucson, AZ, United States; ^5^Department of Sociology, College of Social and Behavioral Sciences, University of Arizona, Tucson, AZ, United States

**Keywords:** Mexican immigrant, OTC medication, demographic factors, sociodemographic, self-medication

## Abstract

**Introduction:**

Over-the-counter (OTC) medication use is high among Mexican immigrants before migrating to the U.S. However, changes in OTC medication use during migration process remain unclear. This study describes OTC medication use patterns among Mexican immigrants in Southern Arizona, explores changes caused during the migration process, and examines the influence of sociodemographic factors on OTC medication use.

**Methods:**

A cross-sectional study was conducted using a purposive community-based sample (*n* = 300) of Mexican immigrant adults. Poisson regression model was utilized to assess the relationship between perceived discrimination, importance of health care use, pre-existing chronic health conditions, years living in the U.S. and the number of OTC medications used in the U.S. while adjusting for demographics and number of OTC medications used in Mexico.

**Results:**

Acetaminophen, NSAID, and supplement use increased post-migration. The number of OTC medications used in Mexico was associated with OTC medication use in the U.S. (RR = 1.35, 95% CI: 1.26, 1.45). Men in the U.S. had a 24% lower rate of OTC medication use compared to women (RR = 0.76, 95% CI: 0.59, 0.97). Length of stay in the U.S. was significantly associated with OTC medication use (95% CI: 0.00, 0.38; *p* = 0.002). Divorced/separated individuals had a 29% lower rate of OTC use compared to married individuals (RR = 0.71, 95% CI: 0.53, 0.96).

**Discussion:**

This study is among the first to examine OTC medication use patterns among Mexican immigrants in Southern Arizona. Further research is needed to understand the factors driving these changes and their impact on health outcomes.

## Introduction

Over-the-counter (OTC) medications, or those that can be purchased without a prescription, are used by approximately 81% of the United States (U.S.) population. The sale of these products contributes to an annual consumer spending of $30 billion (1). When used as directed, OTC medications are considered safe and can yield positive health outcomes by affording relief for minor ailments and contributing to self-care decision-making (2–4). However, when misused, they can have adverse side effects, interact with other medications, and exacerbate medical conditions (5). The misuse of OTC medications contributes to 178,000 hospitalizations annually (6). Although there are no precise estimates for OTC medication use based on racial or ethnic background, available evidence suggests that self-medication practices are highly prevalent among immigrants in the US (7–9).

In Southern Arizona, OTC usage among Mexican immigrants is an integral part of a wider umbrella of self-care behaviors that are practiced at very high rates on both sides of the U.S.-Mexican border (10, 11). Existing evidence among Mexicans while residing in Mexico shows that patterns of OTC usage reflect socio-demographic characteristics that are correlated to healthcare barriers, such as limited access to healthcare, cultural perceptions, documentation status, and low socioeconomic status (12–14). Upon arriving in the U.S., Mexican migration historically followed a circular pattern, meaning many migrants returned home regularly. However, stricter border enforcement in the 1980s and 1990s made returning home difficult and costly, leading many Mexican immigrants to settle permanently in the U.S. (14). Those who established themselves in the new country faced challenges such as exclusion from insurance programs, fear of deportation, and systemic discrimination linked to their documentation status, which collectively reduced their ability to access necessary medical care (8, 15). Mexican immigrants continued practicing health behaviors acquired in their home country, while undergoing a process of cultural and psychological change known as acculturation (16–20).

Recognizing how individuals identify ethnically within this group is essential, as ethnic identity influences social and political behaviors and attitudes (10). Further, there is a common belief among Mexicans that healthcare facilities in Mexico lack the resources necessary to provide adequate care to patients (13). This perception may lead Mexicans to rely on OTC medications as alternative strategies for treatment (13, 21). In Mexican culture, family is highly valued, often leading individuals to prioritize their family’s financial needs over their own health, which may in turn encourage a preference for self-medicating with OTC medications as a form of cost savings (17, 22, 23). During this process, Mexican immigrants, may adopt attitudes, values, and beliefs of U.S. culture, which may influence health behavior changes. Low acculturation, or less adoption of the host culture’s norms and practices, is associated with increased use of OTC medications in the U.S. within the Hispanic population (18). Additional evidence suggests that the level of perceived discrimination and exclusion in the new country may lead to a preference for self-care practices using OTC medications rather than seeking medical care (18). In addition, OTC medication use among Mexican immigrants in the U.S. has been linked to limited access to healthcare services, skepticism of professional healthcare, and preference for self-medication practices with OTC medications (13, 16, 21, 24). All these factors may limit the ability of individuals to access quality healthcare services, thus leading to a reliance on self-medication use (16, 23, 25).

Although OTC medication use is an important self-care strategy that has filled in gaps in healthcare access among Mexicans in the U.S., research indicates that Mexican immigrants with low income and low educational attainment are more likely to misuse OTC medications. This is an issue of particular urgency along the US-Mexico border, where immigrants are more likely to experience low socioeconomic status than the national average and their respective state populations (7, 18, 26). Residents of this area are more likely to obtain non-prescription medication without consulting a doctor due to its affordability and accessibility (18). The fact that self-medication with OTCs has become more widespread in the border region is likely a strategy to alleviate healthcare costs and other barriers, including hesitancy going to the doctor due to immigration (18, 27).

Although many studies have provided insights into patterns of self-medication practices among the Hispanic community broadly, there is a lack of research exploring the use of OTC medications among Mexican immigrants pre- and post-migration to the U.S., and in particular in the border region (16, 28). This is particularly important since the Mexican immigrant community in the U.S. is characterized by a significant population flow that settles in specific areas, have a family-based immigration focus, and challenges such as language barriers and documentation status issues that makes it of public health importance. Many Mexican immigrant families face challenges understanding complex, English-language medication labels. The lack of familiarity with OTC medication practices in the U.S., combined with language barriers, can lead to dosing errors (1, 14). Using a cross-sectional study design, we aim to describe the patterns of OTC medication use among Mexican immigrants in Southern Arizona. Our objective is to determine self-medication patterns and behaviors among Mexican immigrants in Southern Arizona and to identify key factors influencing these behaviors post-migration.

## Methods

### Source of data and study population

A secondary data analysis of Salud sin Fronteras (SSF), a purposive community-based survey conducted between April 2022 and February 2023 to explore barriers to healthcare pre- and post- migration among Mexican immigrants in Southern Arizona, was conducted. Details regarding study procedures have been published elsewhere (8, 24). Briefly, participants were recruited from community-based settings in Southern Arizona. Individuals were eligible to participate if (1) were Mexican-born, (2) migrated from Mexico to the US at least at 15 years of age, (3) resided in Southern Arizona, and (4) were at least 18 years of age. Participants completed a telephone survey, which was proctored by trained bicultural and bilingual research assistants to allow participants to ask clarification questions throughout the data collection process.

### Variables of interests

#### Number OTC medication use

The outcome variable of interest is the number of OTC medications used post-migration, or the period starting from the participant’s settlement in the U.S., derived from the questions: (1) “*Since migrating to Arizona, have you ever self-medicated with commercial or natural products from the pharmacy that were NOT prescribed by a doctor to promote health or treat any ailments, or not?*” categorized as 0 = No, 1 = Yes (8) and (2)*“Number of OTC medications when living in Arizona.”* Participants were given up to five open-ended spaces to report OTC medications. Responses were described as a count variable (range 0–5) (8, 24).

#### Classification of OTC medications

We categorized reported OTC medications into 10 predefined groups based on their active ingredients and therapeutic purposes including (1) acetaminophen, (2) antacids, (3) antidiarrheals, (4) antihistamines, (5) antitussives, (6) compound drugs, (7) homeopathic, (8) NSAID, and (9) supplements. Two additional categories that did not fit in the previous predefined categories were created including: (10) other and (11) unknown. See Table 1 for a detailed description of our classification scheme. Antibiotics were reported by study participants and included in the study due to their therapeutic relevance and the public health implications of their use. For simplification purposes, the antibiotics category was kept in Table 1 to show all reported medications together. However, we recognize that this classification does not align with regulatory definitions and represents a limitation of our study. However, we recognize that this classification does not align with regulatory definitions and represents a limitation of our study.

**Table 1:** Classification of over-the-counter (OTC) medications^1^.

Category	Description	OTC medication responses
Acetaminophen	Active ingredient that relieves pain and fever	Acetaminophen, paracetamol, Tylenol
Antacids	Active ingredient that contains at least 25% of acid neutralizer, and that does not include a laxative or constipating effect	Calcium carbonate/magnesium hydroxide, omeprazole
Antibiotics	A type of antimicrobial agent that works by killing or slowing of bacteria responsible for causing infection.	Penicillin such as amoxicillin and ampicillin
Antidiarrheals	Active ingredient to treat or control diarrhea	Loperamide, bismuth subsalicylate
Antihistamines	Active ingredients used primarily to counteract the effects of histamine, one of the chemicals involved in allergic reactions.	Loratadine, diphenhydramine, dimenhydrinate
Antitussives	Active ingredients that alter the consistency or production of phlegm, such as mucolytics, expectorants, and suppression of coughing reflex; those containing antihistamine were excluded from this category.	Quaifenesin, ambroxol, syrups for cough
Compound drugs	Combined, altered or mixed active ingredients to create a medication tailored to the individual need of an individual.	NSAID, acetaminophen, caffeine, antitussive, antibiotic, antacid, opioid, antidiarrheal
Homeopathic	Natural products including those ingredients derived from plants.	Menthol, honey, herb pills, herb tea, “Vicks” (elderberry, Marshmallow Root & Ivy Leaf).
NSAID	Non-steroidal anti-inflammatory active ingredients to relieve fever, pain and inflammation.	Ibuprofen, naproxen, aspirin
Supplement	Any containing a dietary ingredient intended to supplement the diet, including vitamins and minerals	Vitamin A, B12, C and D, magnesium, Omega 3 and 9, Iron, folic acid, carnitine, calcium, phosphorus
Other*	Any other OTC medications reported by participants that do not fit into the above categories	Antifungal (Tolnaftate 1%.), Antispasmodic (pinaverium bromide), Antiseptic (hydrogen peroxide), Merthiolate, diuretic (Metazolone), corticosteroid (Cortisone), hormone (estradiol).
Unknown*	Any medications that participants could not recall or specify at the time of the phone interview.	Unspecified medications, responses “pills for fever” or “pills for diarrhea”

Categorization based on Food and Drug Administration (FDA) definitions.

^*^Categories created by research team.

^1^Reflects the number and percentage of participants answering ‘yes’.

#### Sociodemographic factors

Demographic variables considered for these analyses were age at migration and screening, education attainment, employment, monthly income, marital status, health insurance, and country of origin. Influential covariates identified *a priori* are frequency of travel to Mexico since living in the U.S., documentation status, and English fluency. Length of stay in the U.S. derived from the question “*In all, how long have you lived or worked in the United States throughout your entire life?”* described as a continuous variable (range 0–55 years). Demographic variables included for the analyses of this study included those who characterize the social determinants of health for every population. Further, the length of stay in the U.S. has been used in migration studies to assess acculturation and its impact on health behaviors and health outcomes (22).

Pre-existing chronic health conditions known pre-and post-migration were derived from the questions “*Did you have any known chronic health conditions/illnesses when you were living in Mexico?*” and “*Have you been diagnosed with any chronic health conditions/illness since you settled in the United?” respectively* and categorized as 0 = No, 1 = Yes. Research indicates that people with chronic conditions often rely on self-medication to alleviate their symptoms (5).

Perceived importance of health care use to be healthy was derived from the question “*Do you currently believe while living here in AZ it is important for you and your family to access medical care?”* categorized as 1 = Yes, 2 = No.

Perceived discrimination was derived from the question “*Have you ever experienced discrimination or unfair treatment when seeking or attempting to seek formal medical care in Arizona?*” was used to assess participants’ experiences with discrimination when seeking healthcare in the U.S. Response options included 0 = No, 1 = Yes.

The perceived importance of healthcare use and perceived discrimination in this study is justified by extensive research documenting how healthcare access, trust, and perceived need for medical services influence healthcare utilization, especially among minority populations, consequently relying on self-medication practices with OTCs (10, 13, 16, 18, 19, 21, 24).

### Statistical analysis

Data preparation and analyses were conducted using R Statistical Software (29). Descriptive statistics were calculated for all substantive and covariate (control) variables. Mean and standard deviations (SD) were calculated for continuous variables, while basic frequencies were used to describe categorical variables.

To test hypotheses, a series of Poisson regressions with robust standard errors were estimated to understand the relationship between sociodemographic factors and the number of OTC medications used in the U.S. All models included the following covariates: OTC medications used in Mexico, age, sex, marital status, educational attainment, and English fluency, and U.S. health insurance coverage to account for potential confounding and to isolate the direct relationship between the sociodemographic variables of interest and the number of OTC medications used in the U.S. Other covariates considered included monthly income, age at migration, documentation status, and health insurance in Mexico. These additional factors were not included in final models as they did not enhance the overall model fit, as determined by chi-square goodness of fit model comparisons. See Supplementary Table 1 for model comparison statistics. To avoid overfitting, a model of the covariates was estimated first, then substantive predictors including diagnoses of chronic disease in Mexico, U.S., discrimination when seeking healthcare in Mexico and the U.S., perceived importance to visit a physician in Mexico and the U.S., and length of stay in the U.S. were estimated individually, one at a time. All estimates from Poisson regressions were computed using robust standard errors to account for any minor deviations from distributional assumptions (11, 30).

## Results

### Participant characteristics

A total sample of 301 participants completed the study. Participants had a mean age of 49.5 years (SD = 13.0) with 41.5% of participants falling between the ages of 35 and 49, while 59.5% immigrated to the U.S. between the ages of 18 and 34. Most participants were female (77.4%), married (71.4%), employed (50.2%), and had a monthly household income between $2,000 and $2,999 (34.1%). In addition, 29.6% of participants completed less than high school and 60.7% spoke English at some level. The average number of OTC medications used while living in Mexico was 1.5 (SD = 1.3), while in the U.S. this average was reported as 1.3 (SD = 1.2). A larger proportion of participants reported a chronic disease diagnosis while living in the U.S. (35.1%) than in Mexico (13.6%). Likewise, more participants reported believing that visiting the physician is important to be healthy while living in the U.S. (98.7%) than in Mexico (76.1%). Most of the sample (83.9%) reported no healthcare discrimination in the U.S. and had an average length of stay in the U.S. of 17.9 years (SD = 11.9). Table 2 contains descriptive statistics for the sample including demographic and all substantive study variables.

**Table 2:** Demographics of study population.

Characteristic	*N* = 301^1^
Age	
18–34 Years	34 (11.3%)
35–49 Years	125 (41.5%)
50–64 Years	97 (32.2%)
65 + Years	45 (15.0%)
Age at Migration	
<18 Years	33 (11.0%)
18–34 Years	179 (59.5%)
35–49 Years	63 (20.9%)
50–64 Years	21 (7.0%)
65 + Years	5 (1.7%)
Sex	
Female	233 (77.4%)
Male	68 (22.6%)
Education	
Less than High School	89 (29.6%)
High School or Equivalent	81 (26.9%)
Some College	45 (15.0%)
College Degree or Higher	86 (28.6%)
Born in Mexico	
Border State	228 (75.7%)
Non-Border State	73 (24.3%)
Employed^2^	151 (50.2%)
Monthly Income	
$0–$999	37 (13.4%)
$1,000–$1,999	70 (25.4%)
$2,000–$2,999	94 (34.1%)
$3,000–$3,999	33 (12.0%)
$4,000+	42 (15.2%)
Marital Status	
Single	36 (12.0%)
Married/Civil Union	215 (71.4%)
Divorced/Separated/Widowed	50 (16.6%)
English Fluency	
None	118 (39.0%)
Not very well	55 (18%)
Well	72 (24%)
Very well	36 (12%)
Fluently	19 (6.3%)
Chronic Disease Diagnosis in U.S.	105 (35.1%)
Chronic Disease Diagnosis in MX	41 (13.6%)
Healthcare Discrimination in U.S.	48 (16.1%)
Believed visiting the physician is important to be healthy while living in U.S.	297 (98.7%)
Believed visiting the physician is important to be healthy while living in MX	229 (76.1%)
Length of stay in U.S.	17.9 (11.9)
OTC Count MX	1.5 (1.3)
OTC Count US	1.3 (1.2)

^1^Mean (SD); *n* (%).

^2^Reflects the number and percentage of participants answering ‘yes’.

### OTC medication use pre- and post-migration

Table 3 contains detailed pre- and post-migration OTC medication use statistics among study participants (31). Pre-migration, acetaminophen was the OTC medication category with the highest proportion of use (29.6%), followed by NSAIDs (22.3%) and antibiotics (21.9%). Post-migration, acetaminophen remained the highest reported (34.2%), followed by NSAIDs (26.9%) and supplements (8.6%). In addition, the data indicate shifts in OTC medication use among Mexican immigrants before and after migration. Acetaminophen and NSAID use increased post-migration. In contrast, antibiotic use decreased from 21.9 to 7.6%, while antacid use fell from 4.98 to 2.3%, and homeopathic remedy use dropped from 9.6 to 5.98%. The use of supplements increased from 4.7 to 8.6%. Additionally, the proportion of participants using OTC medications categorized as “Other” and “Unknown” decreased from 3.99% vs. 0.67 and 3.99% vs. 1.99%, respectively.

**Table 3:** OTC medication utilization among Mexican immigrants pre and post migration.

Characteristic	Pre-Migration (*N* = 301)	Post-Migration (*N* = 301)
OTC Medication		
Acetaminophen	89 (29.6%)	103 (34.2%)
Antacid	15 (4.98%)	7 (2.3%)
Antibiotic	66 (21.9%)	23 (7.6%)
Antidiarrheal	15 (4.98%)	11 (3.7%)
Antihistamine	16 (5.3%)	17 (5.6%)
Antitussive	24 (7.9%)	13 (4.3%)
Compound Drug	33 (10.96%)	25 (8.3%)
Homeopathic	29 (9.6%)	18 (5.98%)
NSAID	67 (22.3%)	81 (26.9%)
Supplement	14 (4.7%)	26 (8.6%)
Other	12 (3.99%)	2 (0.67%)
Unknown	12 (3.99%)	6 (1.99%)

### Sociodemographic variables and post-migration OTC medications

To avoid overfitting, a model of the covariates was estimated first, then substantive predictors including diagnoses of chronic disease in Mexico, U.S., discrimination when seeking healthcare in Mexico and the U.S., perceived importance to visit a physician in Mexico and the U.S., and length of stay in the U.S. were estimated individually, one at a time.

#### Covariate model

In a model of only covariates, sex was associated with the number of OTC medications used in the U.S., *X^2^* (1, 280) *=* 4.7, *p* = 0.031, such that men reported lower medication use *b* = −0.27, *se* = 0.13, 95% confidence interval (CI) (−0.63, −0.04), *p* = 0.03, when compared with women. That is, compared with women, the rate ratio (RR) for men using OTC medications in the U.S. decreased by a factor of RR = 0.76, 95% CI (0.59, 0.98). Marital status was also associated with the number of OTC medications used in the U.S. *X^2^* (2, 278) *=* 7.20, *p* = 0.028, such that divorced or separated individuals reported lower medication use, *b* = −0.34, *se* = 0.15, 95% CI (−0.63, −0.04), *p* = 0.027, when compared with those who were married. Specifically, the rate ratio for separated or divorced individuals decreased by a factor of RR = 0.72, 95% CI (0.53, 0.96). As we expected, the number of OTC medications used in Mexico was significantly associated with the number of OTC medications used in the U.S., *b* = 0.30, *se* = 0.04, 95% CI (0.23, 0.37), *p* < 0.0001. More plainly, with a one unit increase in the number of OTC medications taken in Mexico the rate ratio increased by a factor of RR = 1.35, 95% CI (1.26, 1.45).

#### Sociodemographic models

Regarding predictors of interest, the number of years an individual has lived in the U.S. was significantly associated with the number of OTC medications individuals reported taking in the U.S., *b* = 0.02, *se* = 0.005, 95% CI (0.00, 0.02), *p =* 0.002, while holding all covariates constant. More plainly, with a one unit increase in years lived in the U.S., the rate ratio increased for individuals by a factor of RR = 1.02, 95% CI (1.005, 1.02). No other predictors of interest were associated with the outcome. See Table 4 for estimates.

**Table 4:** Estimates from 6 models assessing the association between sociocultural variables and the number of OTC medications in the U.S.

	Model 1			Model 2			Model 3			Model 4			Model 5			Model 6		
	(*N* = 292)			(*N* = 294)			(*N* = 291)			(*N* = 294)			(*N* = 294)			(*N* = 294)		
	*b*	*se*	95% CI	*b*	*se*	95% CI	*b*	*se*	95% CI	*b*	*se*	95% CI	*b*	*se*	95% CI	*b*	*se*	95% CI
Sociocultural variables																		
Chronic Disease Diagnosis in U.S.	0.14	0.12	(−0.09, 0.36)															
Chronic Disease Diagnosis in Mexico	–	–	–	0.07	0.17	(−0.25, 0.40)												
Healthcare Discrimination in the U.S.	–	–	–				−0.09	0.13	(−0.35, 0.17)									
Believed visiting the physician is important to be healthy while living in U.S.	–	–	–							0.05	0.23	(−0.40, 0.50)						
Believed visiting the physician is important to be healthy while living in Mexico	–	–	–										0.09	0.13	(−0.15, 0.34)			
Length of Stay in the U.S.	–	–	–													0.02**	0.005	(0.006, 0.02)
Covariates																		
OTC Medication Use in Mexico	0.30***	0.04	(0.23, 0.37)	0.30***	0.04	(0.23, 0.37)	0.30***	0.04	(0.23, 0.37)	0.30***	0.04	(0.23, 0.37)	0.30***	0.04	(0.23, 0.37)	0.31***	0.04	(0.24, 0.38)
Age^1^	−0.02	0.02	(−0.07, 0.03)	−0.01	0.02	(−0.06, 0.03)	−0.01	0.02	(−0.06, 0.03)	−0.01	0.02	(−0.06, 0.03)	−0.01	0.02	(−0.06, 0.03)	−0.05	0.03	(−0.10, 0.00)
Sex^2^ (Female)	−0.27*	0.13	(−0.52, −0.02)	−0.27*	0.13	(−0.52, −0.02)	−0.30*	0.13	(−0.55, −0.04)	−0.27*	0.13	(−0.52, −0.03)	−0.28*	0.12	(−0.52, −0.03)	−0.27*	0.13	(−0.52, −0.03)
Education (Less than high school)																		
High School or Equivalent	−0.18	0.14	(−0.46, 0.10)	−0.22	0.14	(−0.50, 0.07)	−0.20	0.15	(−0.49, 0.08)	−0.21	0.14	(−0.49, 0.08)	−0.21	0.14	(−0.49, 0.07)	−0.19	0.14	(−0.47, 0.09)
Some College	0.01	0.18	(−0.35, 0.36)	0.01	0.18	(−0.34, 0.36)	0.03	0.18	(−0.32, 0.38)	0.01	0.18	(−0.35, 0.36)	0.01	0.18	(−0.35, 0.34)	0.06	0.18	(−0.29, 0.41)
College Degree or Higher	−0.01	0.16	(−0.33, 0.30)	−0.05	0.15	(−0.36, 0.25)	−0.05	0.16	(−0.36, 0.27)	−0.05	0.16	(−0.36, 0.27)	−0.05	0.16	(−0.36, 0.24)	0.05	0.16	(−0.26, 0.36)
Marital Status (married/civil union)																		
Single	−0.31	0.22	(−0.74, 0.12)	−0.34	0.22	(−0.77, 0.09)	−0.33	0.22	(−0.75, 0.09)	−0.34	0.22	(−0.77, 0.09)	−0.35	0.22	(−0.78, 0.08)	−0.30	0.21	(−0.71, 0.10)
Divorced/Separated or Widowed	−0.34*	0.15	(−0.63, −0.04)	−0.34*	0.15	(−0.64, −0.04)	−0.35*	0.15	(−0.65, −0.05)	−0.34*	0.15	(−0.63, −0.04)	−0.34*	0.15	(−0.63, −0.04)	−0.34*	0.15	(−0.64, −0.04)
English fluency (How well do you speak English? None)																		
Not very well	0.15	0.14	(−0.13, 0.42)	0.15	0.14	(−0.13, 0.44)	0.15	0.15	(−0.14, 0.43)	0.15	0.14	(−0.13, 0.43)	0.16	0.14	(−0.12, 0.44)	0.12	0.14	(−0.16, 0.40)
Well	−0.02	0.15	(−0.32, 0.28)	−0.01	0.15	(−0.30, 0.29)	−0.02	0.15	(−0.32, 0.28)	−0.02	0.15	(−0.32, 0.29)	−0.02	0.15	(−0.32, 0.28)	−0.04	0.15	(−0.34, 0.25)
Very Well	0.14	0.19	(−0.23, 0.51)	0.15	0.18	(−0.21, 0.51)	0.17	0.18	(−0.19, 0.53)	0.14	0.19	(−0.24, 0.52)	0.15	0.19	(−0.21, 0.52)	0.03	0.19	(−0.33, 0.40)
Fluently	0.45*	0.19	(0.06, 0.84)	0.49*	0.19	(0.12, 0.87)	0.50*	0.19	(0.11, 0.88)	0.48*	0.19	(0.09, 0.87)	0.46*	0.20	(0.09, 0.87)	0.34	0.21	(0.08, 0.75)
U.S. Health Insurance (yes)	0.10	0.11	(−0.12, 0.32)	0.08	0.11	(−0.14, 0.30)	0.10	0.11	(−0.12, 0.32)	0.09	0.11	(−0.13, 0.30)	0.09	0.11	(−0.13, 0.30)	0.14	0.11	(−0.08, 0.36)

se = standard error; CI = confidence interval; * *p* < 0.05; ** *p* < 0.01; *** *p* < 0.001.

^1^Age is divided by 5 to indicate a one-unit increase in age as equal to a 5-year increase in age.

^2^Sex assigned at birth.

## Discussion

The use of OTC medications is a significant yet underexplored aspect of health practices among Mexican immigrants in the U.S., particularly in regions along the U.S.-Mexico border. In this study, we aimed to help fill this gap by examining the patterns of OTC medication use among this population in southern Arizona, both before and after migration, as well as the influence of sociodemographic factors on this practice. Study findings revealed shifts in OTC medication use post-migration, with an increase in the use of acetaminophen, NSAIDs, and supplements. These data point to a need to consider the cultural and socioeconomic contexts of Mexican immigrants to mitigate potential health risks associated with OTC medication use.

Study findings suggest that OTC medication usage patterns while in Mexico persist in the U.S., with factors such as the length of stay in the U.S. influencing OTC medication use. For instance, the longer an immigrant has lived in the U.S., the more OTC medications they reported using. Several factors may explain this trend, including high healthcare costs, lack of health insurance, acculturation, the characteristics of individuals using OTC medications, and the nature of interactions between healthcare providers and users that influence how these medications are utilized (18, 32, 33).

In addition to these findings, sociodemographic factors such as sex and marital status were significant predictors of OTC medication use. In this study, men and divorced or separated individuals reported lower OTC use than women and married individuals. This finding may reflect differences in health-seeking behaviors and social support systems. As seen in the literature, men often have different health-seeking patterns than women. For instance, broadly speaking, men engage less in all types of self-care practices than women, which can result from cultural norms (9, 11). Additionally, in Mexican culture, family is highly valued, and self-care practices are shared among family members as part of cultural norms (19, 20). This cultural norm can also explain disparities in OTC medication use between divorced or separated individuals and married individuals. While this pattern had been explored in the literature, future research should analyze the relationship between cultural norms and gender-specific characteristics that influence health-seeking behaviors in Mexican immigrant populations.

In this study, the perceived importance of visiting a physician in Mexico, the presence of chronic conditions in the U.S., or experiencing discrimination when seeking healthcare were not significantly associated with the number of OTC medications used. The absence of this association could be due to characteristics like health literacy and education level. Health literacy, defined as an individual’s capacity to access, comprehend, and apply health information, is an important consideration in understanding health behaviors (9, 34). While low health literacy has been linked to increased self-medication, particularly among individuals who may not fully grasp the importance of seeking medical care, it is crucial to acknowledge that health-related decision-making among Mexican immigrants is multifactorial. Beyond literacy in the traditional sense, broader health-related knowledge, cultural beliefs, and preferences significantly shape healthcare utilization and medication choices (5, 34). Further, paradoxically, high health literacy levels might relate to greater perceived discrimination (35, 36). For example, higher educational attainment migrants perceive more discrimination in healthcare settings compared to those with lower educational attainment (35, 36). In this study, the largest proportion of participants had less than a high school diploma (29.6%). Therefore, those with lower educational attainment may not recognize or report discrimination in healthcare settings due to a lack of awareness or lower expectations of the quality of healthcare (35, 36). Further research is needed to explore the overlap between health literacy, perceived discrimination, and OTC medication use among migrant populations, specifically Mexican immigrants with different educational levels.

The study highlights changes in OTC medication use post-migration. The higher proportion of acetaminophen (34.2%) and NSAID (26.9%) use in the U.S. compared to that reported in Mexico, 29.6% vs. 22.3% respectively, suggests a potential change in pain management preferences, availability, as well as changes in participants’ health status. Changes in health outcomes are expected and can be attributed to high rates of ecologic stressors post-migration and added barriers to healthcare access. Additionally, as populations age, there is a significant rise in the prevalence of chronic diseases (e.g., diabetes, hypertension, depression). These conditions require increased healthcare services, rehabilitation, and daily living assistance. However, access to these services can become more challenging when multiple comorbidities are present, as they elevate healthcare costs and exacerbate the health burden (7, 37, 43). Individuals with chronic diseases may become more familiar with managing their health through prescribed and OTC medications, either to alleviate persistent symptoms or due to the need to handle long-term conditions independently (37, 43). Therefore, it is important to consider the potential risks of OTC medication use among older adults with comorbidities, as it may lead to harmful medication interactions if they are managing multiple chronic conditions simultaneously (38).

Moreover, the simultaneous use of folk remedies and OTC medications can interact in ways that may enhance or complicate health outcomes. People may use them together to complement one another, such as combining herbal teas or homeopathic remedies with OTC medications (e.g., painkillers or cold medicines) (39). However, certain folk remedies, particularly herbal ones, may interact with OTC medications, potentially increasing side effects or reducing their effectiveness (39). For example, some herbs can potentiate the effects of medications like NSAIDs, leading to risks such as stomach irritation (40). Finally, relying on both types of treatments may delay seeking professional care for more serious conditions. Clear communication with healthcare providers about all remedies in use is essential to ensure safe and effective care. It is also important to note that chronic diseases often arise from a complex interaction of genetic, environmental, behavioral, and socioeconomic factors. This can particularly affect migrants who may face new environmental exposures and different lifestyles in the U.S. compared to Mexico (45).

This study found that the use of anti-inflammatories and painkillers increased post-migration, while antibiotic use significantly decreased (21.9% vs. 7.6%). Given that 21.9% of participants reported using antibiotics pre-migration and only 7.6% post-migration, this decline suggests a shift in accessibility, potentially reflecting differences in healthcare regulations, availability, and informal access to them across migration contexts. Although antibiotics are typically prescription-only in the U.S., their reported usage in this study highlights possible gaps in healthcare access and variations in regulatory enforcement. Their inclusion in our analysis provides valuable insight into self-medication trends and public health concerns within this demographic. However, we acknowledge that categorizing antibiotics alongside OTC medications may not fully capture the complexities of access and availability in diverse healthcare settings.

Contributing factors that could explain lower levels of antibiotic use in the U.S. compared to Mexico include documentation status and medication regulations. Since the purchase of antibiotics in the U.S. requires a medical prescription, this finding was expected, whereas the absence of medication regulations prior to 2010 in Mexico significantly influenced the higher use of certain OTC medications before migration (26, 27). Over a decade ago, unrestricted regulations led to the highest antibiotic use rate in Mexico (21, 41). It was not until 2010 that the Commission for Health Risk Prevention (COFEPRIS) prohibited the purchase of non-prescription antibiotics in Mexico. Whereas in the U.S., the FDA strictly regulates antibiotics, requiring prescriptions from licensed healthcare professionals (38). While regulating antibiotic use could help control its misuse, a decline in antibiotic usage may suggest barriers to healthcare access in the U.S. Active public health campaigns are crucial for promoting the responsible and safe use of antibiotics and increased regulatory guidelines and initiatives may help reduce antibiotic use.

In addition to this shift in antibiotic use, people living along the border tend to travel to Mexico due to its medication affordability and accessibility, specifically antibiotics. Another possible explanation for the lower proportion of participants reporting antibiotic use in the U.S. can be attributed to documentation status. Approximately one-quarter of the study sample was undocumented, which may have limited their ability to return to Mexico to obtain antibiotics or to have access to U.S.-based medical services (26, 27, 41). Despite efforts to mitigate healthcare disparities among immigrants in the U.S., such as the Affordable Care Act (ACA), the fear of deportation can contribute to seeking healthcare access, leading to reliance on OTC medications (14).

Lastly, despite these regulation efforts, the border region is a major entry point not only for OTC medication smuggling but also for prescription and illicit substances (14, 42). The cross-border movement of these medications complicates efforts to regulate their safe use in this region. In addition to the challenges faced in this geographical area, undocumented immigrants may avoid seeking necessary health services due to fear of deportation or being reported to immigration authorities. Therefore, their decision to actively manage their health increases (14). For instance, through the self-dispensation of OTC medication, motivated by barriers to accessing medical care as well as a preference for maintaining culturally rooted practices (10). However, further research is needed to understand the long-term health impacts of self-dispensed medication, cultural practices in health decision-making, and the effects of immigration policies on healthcare access in the U.S.-Mexico border.

### Strengths

Our study method exhibits diverse strengths. First, cultural competency was present among the study staff who recruited and surveyed participants. Most of the staff members who participated in the data collection phase of the study were fluent in English and Spanish and of Mexican descent. The contribution of bilingual, bicultural research assistants fluent in both English and Spanish enhanced the quality of data collection, allowing participants to engage fully. Second, the survey featured open-ended questions, allowing participants to provide more detailed insights into their specific OTC medication usage, which is not usually captured in survey research. Additionally, our analysis produced a detailed classification of OTC medications based on FDA definitions, giving an in-depth view of medication use before and after migration (44). Finally, the study anticipated potential confounders, including sex, age, health insurance, and English fluency, and adjusted for them in the multivariable model.

### Limitations

Our results should be interpreted in the presence of some limitations. Due to its cross-sectional design, the data were collected at one point in time, which means we cannot assume causation or understand how these trends change over time. However, recognizing the importance of causality helps frame the study’s findings as a foundation for future research. For example, longitudinal designs could further explore causality behind the observed patterns of OTC medication use. In addition to the study design, data drawn from the SSF survey were not changed or modified for this study to maintain data integrity and avoid instrumentation bias, allowing us to preserve the validity of findings in this research study. More specifically, this approach helps prevent distortions in data interpretation that could arise from modification of the study instrument (SSF survey). Additionally, convenience sampling may introduce selection bias, potentially making our sample unrepresentative of the broader Mexican immigrant population. Another major limitation is the potential for recall bias. Participants were asked to remember their experiences before migration to the U.S., which can lead to bias in responses among those with a lengthy stay in the U.S. Another limitation, but an important opportunity for future research, is the lack of information on prescription medicines used among study participants, particularly those who reported a chronic disease diagnosis pre- and post-migration. The fact that participants were responding to an open-ended question about which OTC medications they used also poses certain challenges, because their answers were often broad and generalized. Knowing the exact antibiotics purchased is crucial for interpreting data.

## Conclusion

This study is one of the first to examine OTC medication use patterns among Mexican immigrants in Southern Arizona. The findings contribute to a better understanding of self-medication practices among this population and reveal that patterns of OTC medication use observed in Mexico often persist post-migration. There was an observed increase in the use of acetaminophen, NSAIDs, and supplements post-migration, while the use of antibiotics declined. Sociodemographic factors, such as gender, marital status, and length of stay in the U.S., were significantly associated with the number of OTC medications used. These results suggest that personal characteristics and migration-related experiences may influence self-care behaviors. Further research is needed to explore the implications of these patterns for health outcomes, investigate the various interrelated factors shaping OTC medication use, and examine the specific ailments for which these medications are being used among immigrant populations.

## Data Availability

The raw data supporting the conclusions of this article will be made available by the authors, without undue reservation.
