# Supplementary material for: Over-the-counter medication use among Mexican immigrants in Southern Arizona: a cross-sectional study
Source: Front Public Health. 2025 Aug 6;13:1528486. doi: 10.3389/fpubh.2025.1528486 (PMC12364909; doi:10.3389/fpubh.2025.1528486)
Supplement: Supplementary file 1 [file Table_1.docx]

| Supplementary Table 1. |  |  |
| --- | --- | --- |
| *Chi-square Goodness of Fit Statistics for Covariate Variables Not Included During Model Building* | | |
| Variable | *X^2^(df_between_, df_within_)* | *p*-value |
| Monthly Income | 2.67 (4, 260) | 0.615 |
| Age at Migration | 9.18 (4, 277) | 0.058 |
| Documentation Status | 1.10 (4, 263) | 0.893 |
| Health Insurance in Mexico | 0.04 (1,278) | 0.841 |
| *Note.* Covariates were added to the model in the following order: OTC medication count in Mexico, age, sex, marital status, monthly income, education, English fluency, age at migration, documentation status, health insurance in the U.S., and health insurance in Mexico. Variables shown in this table had a non-significant Chi-square goodness-of-fit test, indicating no significant improvement in model fit when the variable was included. | | |
